# Corpuscular Fragility and Metabolic Aspects of Freshly Drawn Beta-Thalassemia Minor RBCs Impact Their Physiology and Performance Post Transfusion: A Triangular Correlation Analysis In Vitro and In Vivo

**DOI:** 10.3390/biomedicines10030530

**Published:** 2022-02-23

**Authors:** Alkmini T. Anastasiadi, Vasiliki-Zoi Arvaniti, Efthymios C. Paronis, Nikolaos G. Kostomitsopoulos, Konstantinos Stamoulis, Issidora S. Papassideri, Angelo D’Alessandro, Anastasios G. Kriebardis, Vassilis L. Tzounakas, Marianna H. Antonelou

**Affiliations:** 1 Department of Biology, Section of Cell Biology and Biophysics, School of Science, National and Kapodistrian University of Athens (NKUA), 15784 Athens, Greece; alkanast@biol.uoa.gr (A.T.A.); vazoarvaniti@gmail.com (V.-Z.A.); ipapasid@biol.uoa.gr (I.S.P.); 2 Center of Clinical, Experimental Surgery & Translational Research, Biomedical Research Foundation, Academy of Athens (BRFAA), 11527 Athens, Greece; eparonis@bioacademy.gr (E.C.P.); nkostom@bioacademy.gr (N.G.K.); 3 Hellenic National Blood Transfusion Centre, Acharnes, 13677 Athens, Greece; kostas.stamoulis@gmail.com; 4 Department of Biochemistry and Molecular Genetics, School of Medicine, Anschutz Medical Campus, University of Colorado, Aurora, CO 80045, USA; angelo.dalessandro@ucdenver.edu; 5 Laboratory of Reliability and Quality Control in Laboratory Hematology (HemQcR), Department of Biomedical Sciences, School of Health & Welfare Sciences, University of West Attica (UniWA), 12243 Egaleo, Greece; akrieb@uniwa.gr

**Keywords:** transfusion, donor variation, biomarkers, red blood cells, fragility, purine metabolism, energy metabolism

## Abstract

The clarification of donor variation effects upon red blood cell (RBC) storage lesion and transfusion efficacy may open new ways for donor–recipient matching optimization. We hereby propose a “triangular” strategy for studying the links comprising the transfusion chain—donor, blood product, recipient—as exemplified in two cohorts of control and beta-thalassemia minor (βThal^+^) donors (*n* = 18 each). It was unraveled that RBC osmotic fragility and caspase-like proteasomal activity can link both donor cohorts to post-storage states. In the case of heterozygotes, the geometry, size and intrinsic low RBC fragility might be lying behind their higher post-storage resistance to lysis and recovery in mice. Moreover, energy-related molecules (e.g., phosphocreatine) and purine metabolism factors (IMP, hypoxanthine) were specifically linked to lower post-storage hemolysis and phosphatidylserine exposure. The latter was also ameliorated by antioxidants, such as urate. Finally, higher proteasomal conservation across the transfusion chain was observed in heterozygotes compared to control donors. The proposed “triangularity model” can be (a) expanded to additional donor/recipient backgrounds, (b) enriched by big data, especially in the post-transfusion state and (c) fuel targeted experiments in order to discover new quality biomarkers and design more personalized transfusion medicine schemes.

## 1. Introduction

While originally suggested by the middle of the 1960s [1], the concept of donor variation effects, namely, that blood donors’ characteristics, both genetic and environmental, may affect the storability and post-transfusion efficacy of red blood cells (RBCs) [2,3], has only been established in the last decade. In terms of transfusion outcome, blood units from female donors have been proposed to be better for same-sex recipients [4,5], whereas glucose-6-phosphate dehydrogenase (G6PD)-deficient individuals have proven to be inadequate donors [6,7]. Moreover, recovery of RBCs from obese donors seems to be lower in animal models [8], while there have also been concerns about administering blood units from smokers to pediatric patients [9,10].

The unique physiology and RBC storability of these (and many additional) distinctive donor groups may be linked to differential post-transfusion phenotypes. There is an increasing number of studies discussing the importance of linking specific physiological and metabolic characteristics of freshly drawn or stored RBCs with post-transfusion parameters of individual events [11,12,13] to reveal possible biomarkers of good storability or beneficial transfusion combinations and move towards more personalized transfusion schemes. For instance, the osmotic fragility of RBCs in transfusion-mimicking conditions is proportional to that of freshly drawn and stored counterparts [14]. Moreover, both storage and osmotic types of hemolysis have been negatively associated with the recovery of RBCs from obese subjects [8]. In the same context, the baseline levels of ribose phosphate in RBCs from G6PD-deficient donors positively impact their post-transfusion recovery, but storage levels of hypoxanthine have the opposite effect [7], a finding also evident in G6PD-normal donors [15]. Finally, a recent retrospective study linked donor polymorphisms in myosin IXB and hemoglobin alpha 2 with decreased hemoglobin increment post transfusion [13]. Most of the studies either link (a) the donor with the blood unit or the post-transfusion efficacy or (b) the blood unit with post-storage parameters. We hereby propose a more “complete” model to study the transfusion chain, by performing paired donor-blood unit-recipient analyses and trying to link—directly or indirectly—freshly drawn blood characteristics with transfusion outcomes. To better clarify the proposed analysis method, we applied it to RBCs from average control and beta-thalassemia minor donors pre-, during and post-storage, using in vitro and in vivo models of transfusion.

**Figure 1:**
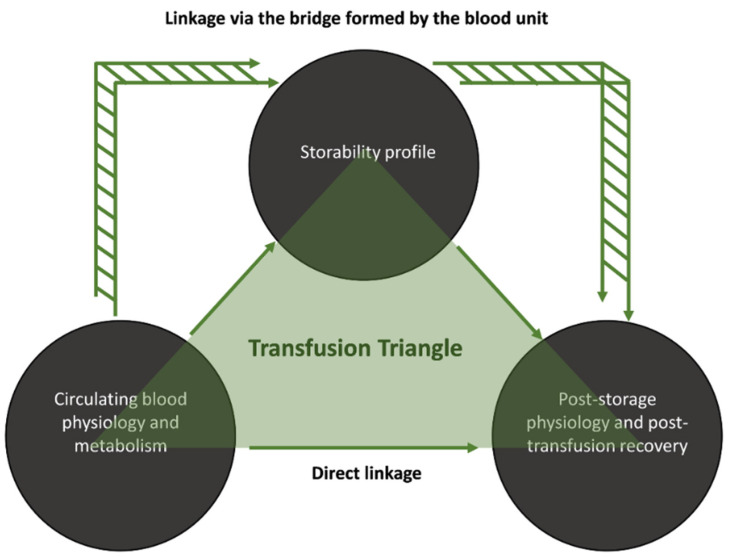
Study design. Proposed routes for the linkage between donor parameters and post-storage/post-transfusion variables.

Circulating RBCs from donors with beta-thalassemia traits (βThal^+^) differ from those of the general population, especially regarding their osmotic fragility and energy metabolism [16]. When stored, βThal^+^ RBCs are resistant to lysis and to membrane/cytoskeleton disruptions and are also able to counteract oxidative and proteotoxic insults [16,17,18]. In the final step of the transfusion chain, βThal^+^ RBCs maintain their resilience against rupture and augmented proteasome activity in an in vitro model of transfusion and they also exhibit a slightly superior recovery in mouse recipients [19]. Since we have already found some statistical links between storage and post-storage phenotypes, we proceeded to search for links between in vivo and (a) storage or (b) post-storage variables. Thus, the aim of the present study was the investigation of any direct or indirect connection between in vivo and post-storage/post-transfusion variables of donated control and βThal^+^ RBCs by bypassing the blood unit or passing through it (Figure 1). Such an analysis will help (a) discover easily accessible candidate biomarkers of post-storage performance and (b) reveal the links between the baseline physiology of βThal^+^ RBCs and their superior post-storage characteristics.

## 2. Materials and Methods

### 2.1. Biological Samples

Freshly drawn blood in citrate vacutainers and leukoreduced units of RBCs stored in citrate–phosphate–dextrose (CPD)/saline–adenine–glucose–mannitol (SAGM) from 18 control and 18 beta-thalassemia heterozygotes were analyzed as previously reported [16,17,18,19] for a significant number (approximately 300 or 700 variables in freshly drawn or stored samples, respectively) of quantitative hematological, biochemical, hemolysis (storage, osmotic, mechanical, oxidative), redox (extracellular antioxidant capacity), metabolome and proteome parameters. Heterozygosity was confirmed by Hb electrophoresis and molecular identification of mutations (including the common Mediterranean mutations IVS I-1, IVS I-6, IVS I-110, IVS II-1 and IVS II-745) [16]. All studies were approved by the Ethics Committee of the Department of Biology, School of Science, NKUA and investigations were carried out with donor consent, in accordance with the principles of the Declaration of Helsinki.

### 2.2. Post-Storage Experiments

Stored RBCs from 10 units per group were reconstituted in plasma from healthy and transfusion-dependent beta-thalassemia major subjects (*n* = 10 per group) in a RBC/plasma volume ratio corresponding to transfusion of two RBC units. Then, the reconstituted samples were incubated for 24 h at body temperature before measuring several hemolysis and redox parameters, as previously reported [19]. Freshly drawn and stored RBCs from the remaining eight donors per group were transfused to immunosufficient (C57BL/6J) and immunodeficient (NOD.CB17-Prkdcscid/J) mice to assess their 24 h recovery [19]. The study was approved by the Department of Agriculture and Veterinary Service of the Prefecture of Athens (Permit Number: 534915, 23 July 2020).

### 2.3. Statistical Analysis

Statistical analysis was performed using the statistical package SPSS Version 22.0 (IBM Hellas, Athens, Greece, administered by NKUA) and significance was accepted at *p* < 0.05 according to the following rationale, graphically presented in Figure 1. Considering that the aim of the present study is to reveal potential linkages between the pre- and post-storage states of RBCs, there are two possible paths to follow, which in combination produce a “transfusion triangle”: (a) the direct one, namely, “pre-storage to post-storage” that bypasses the storage itself and (b) the indirect one, which links freshly drawn blood to storage and subsequently storage to post-storage variables. In the second case, the parameters connecting pre- and post-storage states via the storage bridge had to satisfy the Bonferroni-like adjustment for multiple comparisons. In this context, we examined, for the first time, possible connections between freshly drawn blood and either stored or reconstituted/transfused RBCs. Moreover, we evaluated which of the previously reported storage/post-storage connections [19] satisfy the new statistical criteria. Correlations between freshly drawn, stored and post-storage parameters (including recovery in animal models) were evaluated by the Pearson’s test following examination of the variables for normal distribution and the presence of outliers (Shapiro–Wilk, Kolmogorov–Smirnov tests and detrended normal Q–Q plots). Since Pearson’s test is highly sensitive to outliers, such values were excluded, and the analysis was performed again to minimize the false discovery rate associated with the small size of our groups. If the outcome was not modified, the outlier was included back in the subgroup. In addition, all correlations were also validated by Spearman’s test. Regarding links between non-stored and stored samples, only correlations that were evident at every time-point of storage (weekly measurements) were accepted as reliable. This was also the case for the links between non-stored/stored and post-storage variables, where both time-points (early and late storage) and plasma environments (healthy and beta-thalassemic) were considered, unless otherwise stated.

## 3. Results

### 3.1. Transfusion Triangles Evident in Both Donor Cohorts

**Figure 2:**
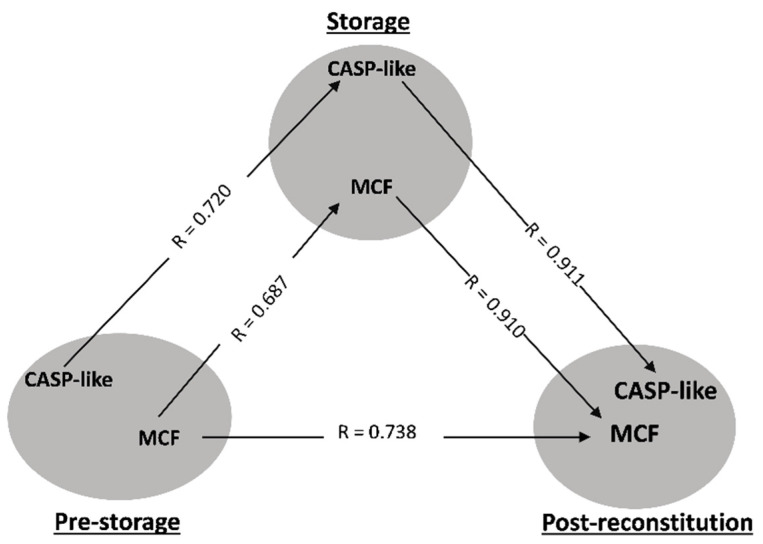
Statistically significant correlations between freshly drawn blood, stored, and reconstituted RBCs from control donors. The correlation triangles focus on osmotic fragility (MCF) and caspase-like (CASP-like) proteasomal activity. All connections represent repeatable (at every recipient plasma and storage time-point measured) significant correlations. The R-values concern late storage and thalassemic plasma.

**Figure 3:**
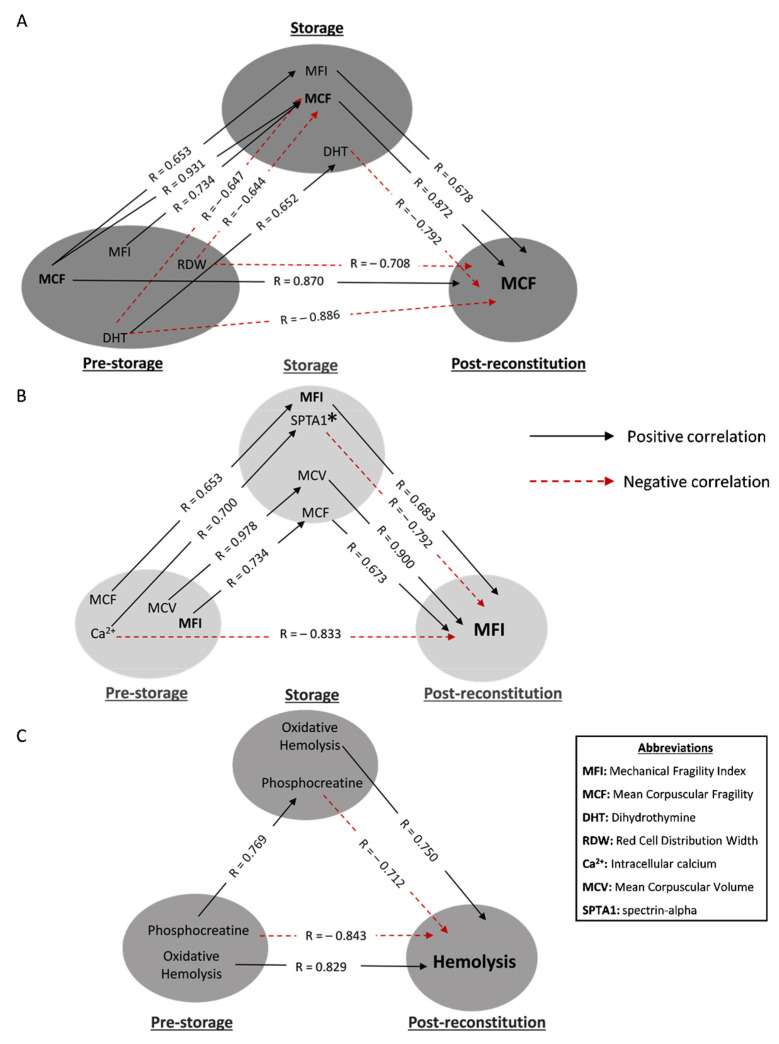
Statistically significant hemolysis correlations between freshly drawn blood, stored, and reconstituted RBCs from beta-thalassemia trait (βThal^+^) donors. The correlation triangles focus on (**A**) osmotic, (**B**) mechanical and (**C**) spontaneous hemolysis of reconstituted samples. All connections represent repeatable (at every recipient plasma and storage time-point measured) significant correlations. The R-values concern late storage and thalassemic plasma. * SPTA1 was used as an example of an array of structural components that correlate with MFI (e.g., ankyrin-1, glycophorin C and 4.2 protein).

**Figure 4:**
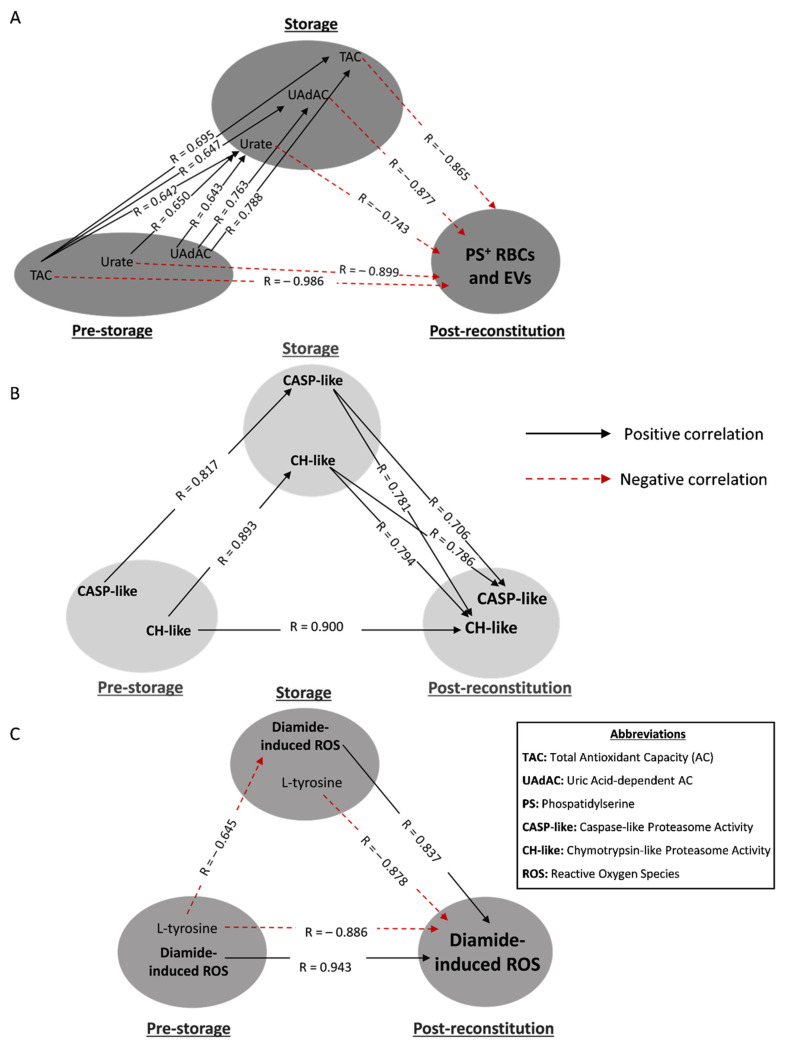
Statistically significant redox-related correlations between freshly drawn blood, stored, and reconstituted RBCs from beta-thalassemia trait (βThal^+^) donors. The correlation triangles focus on (**A**) PS-exposing RBCs and extracellular vesicles (EVs), (**B**) proteasome activity and (**C**) diamide-induced intracellular ROS of reconstituted samples. All connections represent repeatable (at every recipient plasma and storage time-point measured) significant correlations. The R-values concern late storage and thalassemic plasma. Antioxidant capacities of (**A**) are extracellular, while urate is the intracellular metabolite.

In order to determine whether the currently proposed model can provide new information, we applied it to control and βThal^+^ donor groups. The triangularity analysis highlighted two physiological parameters in both cohorts studied: osmotic fragility and caspase-like (CASP-like) proteasomal activity. Regarding osmotic hemolysis, the pre-storage levels were proportional to those of storage and post-storage in vitro states, with an interesting strong intra-correlation linkage present between the stored and reconstituted samples (Figure 2 and Figure 3A). In addition, CASP-like activity during storage was proportional to the one before, and furthermore positively correlated with the post-storage levels in vitro (Figure 2 and Figure 4B).

Besides these two parameters, which formed “complete” (three connections) or “partial” (two connections) transfusion triangles in both the average control and βThal^+^ donors, there were additional variables that satisfied the triangularity criteria in the case of heterozygotes, as described in the following paragraphs.

### 3.2. Hemolysis-Related Transfusion Triangles in βThal^+^

Initially, we focused on transfusion triangles targeting post-storage hemolysis profiles (Figure 3).

The osmotically induced hemolysis of freshly drawn βThal^+^ RBCs positively correlated with the mechanically induced one in the unit, which was in turn linked to osmotic fragility post storage (Figure 3A). The first link was also observed vice versa, i.e., the mechanical fragility pre-storage was associated with the osmotic fragility during storage. Moving on to negative correlations, the red cell distribution width (RDW) index of freshly drawn RBCs was inversely associated with the osmotic hemolysis of both stored and reconstituted samples, as was the case for dihydrothymine, too. This metabolite presented stored levels proportional to the in vivo ones and inversely proportional to the osmotic fragility post reconstitution (Figure 3A).

The mechanical hemolysis of reconstituted samples positively correlated with both the mechanical and osmotic fragility of stored RBCs, as well as with their mean corpuscular volume (MCV), which varied proportionally to the in vivo levels (Figure 3B). An interesting finding was the direct negative link between the baseline levels of intracellular calcium and mechanical hemolysis under in vitro recipient-mimicking conditions. This link was broken in the other two sides of the triangle by (a) a positive correlation of calcium with integral membrane/cytoskeletal proteins of stored RBCs and (b) a subsequent negative correlation of the latter with the mechanical fragility index post storage (Figure 3B).

The levels of spontaneous hemolysis post storage in the same donor group were positively related to the oxidatively induced hemolysis levels of both freshly drawn and stored RBCs (Figure 3C). A “complete triangle” was formed for the following parameter as well: the in vivo and storage levels of phosphocreatine were correlated with each other, and both of them seemed to negatively affect post-storage hemolysis (Figure 3C).

### 3.3. Redox- and Proteostasis-Related Transfusion Triangles in βThal^+^

Concerning the antioxidant power of the RBC units, the levels of total (TAC) and uric acid-dependent antioxidant capacities (UAdAC) of fresh βThal^+^ plasma intra- and inter-correlated with the respective levels in the supernatant, as well as with the intracellular levels of urate in stored RBCs. Moreover, all three storage parameters were negatively associated with the externalization of phosphatidylserine (PS) and the production of PS^+^ extracellular vesicles (EVs) under recipient-mimicking conditions (Figure 4A). Urate also presented an intra-correlation between fresh and stored RBCs. Remarkably, two “complete triangles” were formed since the in vivo levels of both TAC and urate were also directly inversely linked to the PS^+^ RBCs and EVs post storage (Figure 4A).

In the case of proteasome, the (mainly cytosolic) levels of chymotrypsin-like (CH-like) activity were linked with each other in a “complete transfusion triangle” (Figure 4B). Notably, the storage levels of CASP-like and CH-like activities positively correlated with their levels post storage (Figure 4B).

The accumulation of reactive oxygen species (ROS) post stimulation of reconstituted βThal^+^ RBCs with thiol-oxidizing agents (in our case, diamide), was proportional to the in vivo and storage levels (Figure 4C). On the contrary, the levels of L-tyrosine either in freshly drawn RBCs or their stored counterparts seem to be inversely linked to the production of diamide-induced ROS in both stored and reconstituted RBCs (Figure 4C).

### 3.4. Direct Linkages of Freshly Drawn βThal^+^ RBCs to Transfusion Variables In Vitro and In Vivo

**Figure 5:**
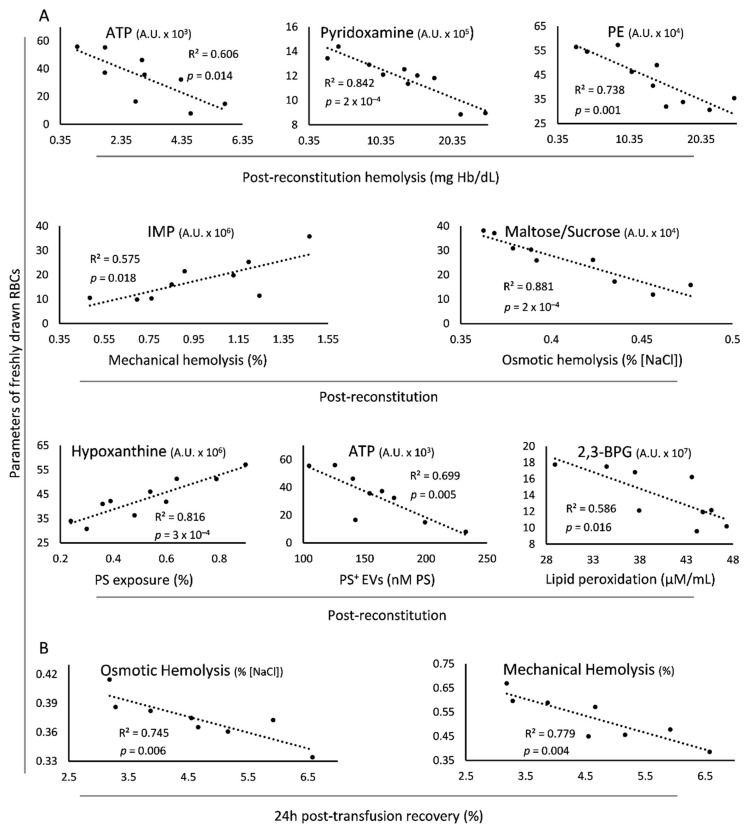
Statistically significant correlations of parameters of freshly drawn blood with the post-reconstitution physiology and 24 h recovery of beta-thalassemia trait (βThal^+^) donated RBCs. (**A**) Baseline levels of metabolites correlating with physiological parameters of reconstituted RBCs. (**B**) Baseline fragility indices correlating with post-transfusion recovery in mice. The selected scatterplots concern late storage, thalassemic plasma (**A**) and C57BL/6J mice (**B**), but significant connections (with slightly different R^2^ values) were evident at every condition tested (i.e., early/late storage, control/thalassemic plasma, immunodeficient/sufficient mice), with the exception of ATP and hemolysis, a correlation evident only in early storage. PE: phosphoethanolamine; 2,3-BPG: 2,3-bisphosphoglycerate; PS: phosphatidylserine; EVs: extracellular vesicles.

Finally, some interesting correlations were evident between freshly drawn and reconstituted/transfused βThal^+^ RBCs, as shown in Figure 5.

The levels of spontaneous hemolysis in the reconstituted samples were negatively associated with those of ATP, pyridoxamine and phosphoethanolamine of freshly drawn RBCs (Figure 5A). Regarding the two RBC fragilities post storage, the mechanical and the osmotic, they were positively or negatively related to the in vivo levels of IMP and maltose/sucrose, respectively. Interestingly, there was a positive correlation of the pre-storage erythrocytic hypoxanthine with the post-storage exposure of PS, along with an inverse linkage between ATP and PS^+^ EVs of the same RBC states. Of note, the in vivo levels of 2,3-bisphosphoglycerate negatively correlated with the magnitude of lipid peroxidation in the reconstituted samples’ membrane (Figure 5A). Some of the above-mentioned correlations (e.g., in vivo levels of ATP and hemolysis post storage (R^2^ = 0.568, *p* = 0.141), in vivo levels of 2,3-BPG and lipid peroxidation post storage (R^2^ = 0.528, *p* = 0.165)) presented the same trend in the control group, too. However, the smaller number of paired control samples compared to the βThal^+^ pairs rendered the statistical analysis highly unsound in this cohort. Last but not least, osmotic and mechanical fragilities of freshly drawn βThal^+^ RBCs exhibited an inverse link with their 24 h recovery in mouse recipients (Figure 5B). In this case, the linkage is strongly related to the physiology of the βThal^+^ RBCs, since it did not emerge in the control cohort which consisted of in vivo vs. transfused RBC pairs equal to βThal^+^ (R^2^ = 0.355 for osmotic and R^2^ = 0.298 for mechanical fragility, *p* > 0.05).

## 4. Discussion

We hereby propose a paired “triangular” model for studying the transfusion chain, presenting it through a correlation analysis of paired donor–storage–post-storage data. Interestingly, out of hundreds of variables tested in freshly drawn blood and stored RBCs, two parameters stood out, fulfilling the triangularity criteria in both the average and genetically distinct βThal^+^ donor groups: osmotic fragility and CASP-like proteasome activity, which have the potential to link donors to post-storage RBC features, at least in vitro. Furthermore, we provide evidence regarding a well-characterized and homogeneous (in terms of RBC geometry, proteostasis and metabolism) donor group, in which a variety of fragility and redox parameters, along with metabolites of purine and energy pathways, seem to be linked directly or indirectly to their overall superior post-storage phenotypes.

The currently proposed triangular approach of transfusion research has the potential to provide reliable and statistically solid links between donors, blood units and post-storage/post-transfusion metrics (probably recipient factors, too). It also highlights the significance of (a) cellular indices that may affect RBC performance, as with the case of sub-lethal storage lesions in βThal^+^ RBCs and (b) direct linking between donor attributes and transfusion outcomes, which presuppose analyses in freshly drawn blood. Regarding the first point, the correlation profile of sublethal defects versus the typical hemolysis metric stands as a representative example. It seems that the multiparametric nature of spontaneous hemolysis [20] does not allow for the completion of a transfusion triangle, while at the same time both osmotic and mechanical fragilities arise as candidate biomarkers of RBC physiology and 24 h post-transfusion recovery in mice. As for the second observation, notable potential links of donor characteristics (including RBC metabolism) with the post-transfusion performance have been lately reported in obese and G6PD-deficient donor groups [7,8]. However, the need for such paired studies cannot a priori bypass the storage factor since the blood unit represents a dynamic rather than a static state (i.e., aging pathways related to both storage and metabolic time) that links donors to recipients in time and space; therefore, it is important to consider both the links between freshly drawn and stored blood [21,22], as well as those between stored and transfused blood [7,19].

Paired analyses, like the one presented in our study, can serve as the first step in elucidating biomarkers of storage and transfusion quality out of a large number of metrics that arise through the implementation of high- or low-throughput techniques. Such studies can feed more targeted experimental approaches in larger cohorts and different donor groups to validate, expand or reject hypotheses that lean on initial observations. Whatever the result of the latter approaches might be, the search for donor/recipient signatures in specific transfusion events will narrow, step by step, the distance between where we currently stand and the much-anticipated future of precise transfusion medicine. Such information can also guide blood logistics strategies in periods of inventory shortage, such as the one imposed worldwide by the COVID-19 pandemic.

The application of the proposed model expanded the previously reported [14] biomarker potential of osmotic fragility since the direct connection between the pre-donation and post-storage levels completed the transfusion triangle in both donor cohorts. However, in terms of post-transfusion performance, it seems plausible that the low susceptibility of βThal^+^ RBCs to both osmotic and mechanical lysis benefits them in the circulation of transfused mice mainly due to their unique geometry and membrane/cytoskeleton proteome [18,23] in comparison to controls. The contribution of cell size and geometry to the mechanical and osmotic stability of stored/reconstituted βThal^+^ RBCs is also hinted at by the presence of hematological indices, such as MCV and RDW in the respective transfusion triangles. The lower volume (MCV: 70.4 ± 5.8 vs. 85.2 ± 4.6 fL, βThal^+^ vs. control in vivo), expressed (directly or indirectly) by the above-mentioned variables, probably protects the cells from stress-induced rupture, a hypothesis consistent with the previously observed positive correlation between RDW and osmotic stability in diabetic samples [24]. The size and shape of these cells might also be one of the reasons behind the special interplay between mechanical and osmotic fragility. It should be noted that the effect of both fragilities (in vivo and during storage [19]) upon recovery emphasizes the importance of sublethal storage lesions in the efficacy of transfusion therapy.

The metabolism of βThal^+^ cells was found to be tightly related to several physiological features. Breakdown intermediates of pyrimidines and purines were found to be “connected” positively or negatively, respectively, to cell fragility and the exposure of removal signals. Focusing on purine metabolism, the negative association between in vivo levels of IMP and mechanical hemolysis post storage came as no surprise in the light of the formerly revealed positive correlations between IMP and storage hemolysis in control samples [25]. In the same context, the observed positive correlation between hypoxanthine and PS externalization, either in cells or vesicles, comes to support the previously shown negative effect of this metabolite upon post-transfusion recovery [15]. Of note, both parameters present lower baseline levels in heterozygotes when compared to controls [16]. High-energy compounds, such as ATP, along with glycolysis intermediates, constitute frequently occurring beneficial biomarkers in transfusion medicine [20,25,26,27], hence their currently observed protective properties with regards to lysis (e.g., in vivo ATP and spontaneous post-storage hemolysis) and oxidative stress-related (e.g., in vivo 2,3-bisphosphoglycerate and post-storage lipid peroxidation) variables in both βThal^+^ and control (though as a trend). This regular emergence of energy-related molecules in such analyses, both ex vivo and in vivo, is consistent with the abundance of volume-regulating channels, membrane-asymmetry maintaining molecules and antioxidants that rely on the energy reservoir of the cells [28]. An interesting observation was the linkage of post-reconstitution βThal^+^ osmotic hemolysis with maltose/sucrose, oligosaccharides known to decrease the membrane lesions on frozen RBCs [29]. Another energy-related molecule, phosphocreatine, was found to anticorrelate with spontaneous hemolysis post-storage, forming another “complete transfusion triangle” in heterozygotes. Apart from providing energy-based support, this molecule is also known to interact with the membrane in a protective manner [30], as previously supported by its negative association with hemolysis in early storage [25].

As in the case of phosphocreatine, the donor levels of antioxidant pyridoxamine—the storage levels of which also anticorrelate with post-storage hemolysis [19]—similarly possesses biomarker potential in βThal^+^ RBCs. Pyridoxamine, which has been found to be elevated in these RBCs [16], plays a significant cytoprotective role by eliminating free radicals and maintaining Na^+^/K^+^-ATPase activity [31]. On the other hand, susceptibility to oxidative lysis, either before or during storage, seems to increase the propensity to spontaneous lysis after exposure of βThal^+^ RBCs to plasma and body temperature. Indeed, hemoglobin oxidation is associated with cellular aging, loss of integrity and, consequently, hemolysis during cryopreservation [32]. In the same context, the interplay between the redox active amino acid L-tyrosine, known for its radical scavenging properties [33], and the generation of ROS with thiol-targeting reagents was rather anticipated.

The most integrated transfusion triangle in our study was the one connecting the antioxidant powers (extra- and intra-cellular) with the post-storage exposure of PS in βThal^+^ RBCs. The mitigation of oxidative stress inside and outside the cell, mediated by non-enzymatic antioxidants, such as urate, appears to be associated with a decrease in PS exposure. The slightly elevated, though within normal range, baseline levels of Ca^2+^ in βThal^+^ cells [16] seemed to affect neither the lipid bilayer asymmetry [34] nor calpain recruitment to the membrane [18] but, on the contrary, they positively correlated to a superior cytoskeleton preservation, “protecting”—directly or not—the mechanical integrity of the cell. Calcium cations participate in a huge variety of intracellular metabolic and signaling networks in RBCs [35], including the reversible loosening of the cytoskeleton during passage through low-diameter capillaries [35] and the tyrosine phosphorylation of Band 3 (increased in stored βThal^+^ membranes [18]) that may energetically support the RBC through unbinding of glycolytic enzymes [36]. Interestingly, a part of the proteostasis of RBCs, the CH-like and CASP-like proteasome activities, is highly preserved both in bag and post storage, highlighting the proteasome as a strong βThal^+^-specific feature in RBCs previously reported to exhibit unique proteo-vigilance abilities during storage [17].

Overall, the application of the currently proposed approach to our donor cohorts provided interesting (either anticipated or biologically reasonable) information and unraveled variables as potential biomarkers of RBC performance. The abundance of transfusion triangles in the group of βThal minor donors reflects the “compactness” of this cohort in regard to several of the currently measured parameters. It may also be associated with the fact that a significant part of the freshly drawn blood variables (e.g., cellular fragilities, intracellular Ca^2+^, pyridoxamine, extracellular antioxidant capacity) that are linked—directly or indirectly—to post-storage physiology or performance differ between βThal^+^ and control donors from the time of donation or during storage, too [16]. On the other hand, there are also parameters, such as proteasome activities, dihydrothymine or phosphocreatine, with control range variation which satisfy the “triangularity criterion” only in βThal^+^ donors, indicating a unique, βThal^+^-related variability. Although it is tempting to hypothesize the presence of thresholds producing differential correlation profiles in distinct genetic subgroups (in our case, βThal^+^ carriers with Hb levels > 13.5 g/dL) the small size of our cohort cannot allow us to draw strict conclusions for such cut-off values.

In conclusion, we strongly believe that the application of the triangularity approach to wide cohorts of average donors and to different donor/recipient backgrounds, as well as its enrichment by omics data (genomics, lipidomics, metabolomics, proteomics, modifomics, etc.) corresponding to RBCs, plasma and extracellular vesicles may lead to the discovery of a significant number of candidate biomarkers for assessing the transfusion quality of RBCs.

## Data Availability

All data presented in this study are available upon request.
